# Supplementary material for: Mechanisms governing the pioneering and redistribution capabilities of the non-classical pioneer PU.1
Source: Nat Commun. 2020 Jan 21;11:402. doi: 10.1038/s41467-019-13960-2 (PMC6972792; doi:10.1038/s41467-019-13960-2)
Supplement: Supplementary file 7 — Source data [file 41467_2019_13960_MOESM7_ESM.zip › Source_Data/FigureS2/FigureS2B_MotifScanOutput/KmeansCluster4/homerResults/motif47.similar.html]

motif47

## Information for motif47

C
G
T
A
T
C
G
A
A
G
T
C
A
T
G
C
C
T
G
A
A
G
T
C
T
G
C
A
  
Reverse Opposite:  

A
C
G
T
T
C
A
G
A
G
C
T
A
T
C
G
T
C
A
G
A
G
C
T
G
C
A
T
  

|  |  |
| --- | --- |
| p-value: | 1e-19 |
| log p-value: | -4.488e+01 |
| Information Content per bp: | 1.662 |
| Number of Target Sequences with motif | 1455.0 |
| Percentage of Target Sequences with motif | 32.23% |
| Number of Background Sequences with motif | 10777.0 |
| Percentage of Background Sequences with motif | 25.78% |
| Average Position of motif in Targets | 99.5 +/- 59.0bp |
| Average Position of motif in Background | 99.8 +/- 67.1bp |
| Strand Bias (log2 ratio + to - strand density) | 0.1 |
| Multiplicity (# of sites on avg that occur together) | 1.19 |
| Motif File: | file (matrix) reverse opposite |

### Similar de novo motifs found

|  |  |  |  |  |  |  |  |
| --- | --- | --- | --- | --- | --- | --- | --- |
| Rank | Match Score | Redundant Motif | P-value | log P-value | % of Targets | % of Background | Motif file |
| 1 | 0.776 | C T G A A G C T A T C G C G A T C A T G C A T G A G C T C A G T C T G A A C T G | 1e-17 | -40.074859 | 6.87% | 3.94% | motif file (matrix) |
| 2 | 0.776 | C G T A A G T C C G T A C G T A A G T C A G T C C G T A | 1e-17 | -39.330408 | 8.08% | 4.91% | motif file (matrix) |
| 3 | 0.859 | G C A T C T G A C T G A A G T C G A T C C T G A A G T C G T C A | 1e-16 | -38.781228 | 10.81% | 7.13% | motif file (matrix) |
